# Association of *TIM-3* with anterior uveitis and associated systemic immune diseases: a Mendelian randomization analysis

**DOI:** 10.3389/fmed.2023.1183326

**Published:** 2023-06-15

**Authors:** Dan Lin, Rong-Cheng Zhu, Chun Tang, Fen-Fen Li, Mei-Ling Gao, Yu-Qin Wang

**Affiliations:** ^1^National Clinical Research Center for Ocular Diseases, Eye Hospital, Wenzhou Medical University, Wenzhou, China; ^2^State Key Laboratory of Ophthalmology, Optometry and Visual Science, Eye Hospital, Wenzhou Medical University, Wenzhou, China; ^3^The Second School of Medicine, Wenzhou Medical University, Wenzhou, China

**Keywords:** *TIM-3*, Mendelian randomization, causal inference, anterior uveitis, Crohn's disease

## Abstract

**Background:**

We aimed to investigate the causal association between *TIM-3*, an immune checkpoint inhibitor, and anterior uveitis (AU), as well as associated systemic immune diseases.

**Materials and methods:**

We performed two-sample Mendelian randomization (MR) analyses to estimate the causal effects of *TIM-3* on AU and three associated systemic diseases, namely ankylosing spondylitis (AS), Crohn's disease (CD), and ulcerative colitis (UC). Single-nucleotide polymorphisms (SNPs) associated with AU, AS, CD, and UC were selected as the outcomes: AU GWAS with 2,752 patients with acute AU accompanied with AS (cases) and 3,836 AS patients (controls), AS GWAS with 968 cases and 336,191 controls, CD GWAS with 1,032 cases and 336,127 controls, and UC GWAS with 2,439 cases and 460,494 controls. The *TIM-3* dataset was used as the exposure (*n* = 31,684). Four MR methods, namely, inverse-variance weighting (IVW), MR-Egger regression, weighted median, and weighted mode, were used in this study. Comprehensive sensitivity analyses were conducted to estimate the robustness of identified associations and the potential impact of horizontal pleiotropy.

**Results:**

Our studies show that *TIM-3* is significantly associated with CD using the IVW method (OR = 1.001, 95% CI = 1.0002–1.0018, *P*-value = 0.011). We also found that *TIM-3* may be a protective factor for AU although these results lacked significance (OR = 0.889, 95% CI = 0.631–1.252, *P*-value = 0.5). No association was observed between the genetic predisposition to particular *TIM-3* and susceptibility to AS or UC in this study. No potential heterogeneities or directional pleiotropies were observed in our analyses.

**Conclusion:**

According to our study, a small correlation was observed between *TIM-3* expression and CD susceptibility. Additional studies in different ethnic backgrounds will be necessary to further explore the potential roles and mechanisms of TIM-3 in CD.

## Introduction

T-cell immunoglobulin and mucin domain (TIM) protein family members have been identified as important regulators of the immune response. TIM-family proteins, which belong to the Type I group of transmembrane proteins, contain an N-terminal variable immunoglobulin (IgV)-like and mucin-like domain in the extracellular region, a single transmembrane domain, and an intracellular domain (1, 2). TIM-3 is a member of the TIM family and functions as an inhibitory costimulatory signal (3, 4). TIM-3, which is found both in T helper 1 (Th1) cells and T helper 17 (Th17) cells, monocytes, and macrophages but not in Th2 cells (3, 5), has been established as a negative regulatory molecule that plays an important role in controlling inflammation (6). Certain pathologies, including autoimmune diseases, infection, allergy, and cancer, have been reportedly associated with TIM-3 dysfunction and genetic polymorphisms (7).

Uveitis contributes to a number of significant, often blinding, ocular inflammation conditions. Anterior uveitis (AU) accounts for the majority of uveitis cases based on surveys from different countries worldwide (8). AU is often linked to seronegative spondyloarthropathies, such as ankylosing spondylitis (AS), reactive arthritis, and psoriatic arthritis, as well as inflammatory bowel disease (IBD), including Crohn's disease (CD) and ulcerative colitis (UC). Despite numerous studies, however, the exact pathogenesis of AU and its associated systemic autoimmune disease (ADs, herein used to refer to AS, CD, and UC) remains unclear. However, it is thought that abnormal activation of both innate and adaptive immune systems, particularly the IL-23/IL-17 axis and Th1/Th2/Th17 effector T-cell lineage, plays a key role (9–12). A meta-analysis of Chinese populations revealed that *TIM-3* polymorphisms are significantly associated with an increased risk of autoimmune diseases, such as rheumatoid arthritis, Graves' disease, multiple sclerosis, systemic lupus erythematosus, AS, CD, and UC (13). Our previous study identified a temporary increase in TIM-3 expression levels in a mouse model of anterior chamber-associated immune deviation (ACAID) (14). Two polymorphisms, *TIM-3* −575G/T and +4259T/G, have been shown to downregulate the gene expression in CD4^+^ T cells, CD8^+^ T cells, and monocytes, thereby increasing susceptibility to AS (15). Furthermore, increased expression of TIM-3 has been found in neutrophils of AS patients compared to healthy controls (16). The proportion of Tim-3^+^ Treg cells is significantly lower in the peripheral blood mononuclear cells (PBMCs) of AS patients, with some of these Treg-mediated functions being less potent (17). Low expression of TIM-3 by Th1 cells and mRNA levels in PBMCs has been found in patients with Crohn's disease (18, 19). In a mouse adoptive transfer model of colitis, a lack of Ceacam1 (carcinoembryonic antigen cell adhesion molecule 1, a ligand of TIM-3) in T cells resulted in a hyper-inflammatory profile with reduced expression of TIM-3 and a decrease in regulatory cytokines (20). These observational and experimental findings suggest that TIM-3 may play important roles in AU and associated ADs even though a causal relationship has not yet been established. For these reasons, it is too early to conduct large-scale randomized controlled clinical trials to evaluate the effects of TIM-3 on AU and related ADs.

Mendelian randomization (MR) is an epidemiological method that uses germline genetic variants as instruments for exposures to infer causality from observational data (21–23). MR analysis provides a feasible and safe way to overcome the limitations of conventional study design. Herein, we used two-sample MR to determine whether *TIM-3* is causally implicated in the onset of AU and associated systemic immune diseases.

## Materials and methods

### Study design

**Figure 1:**
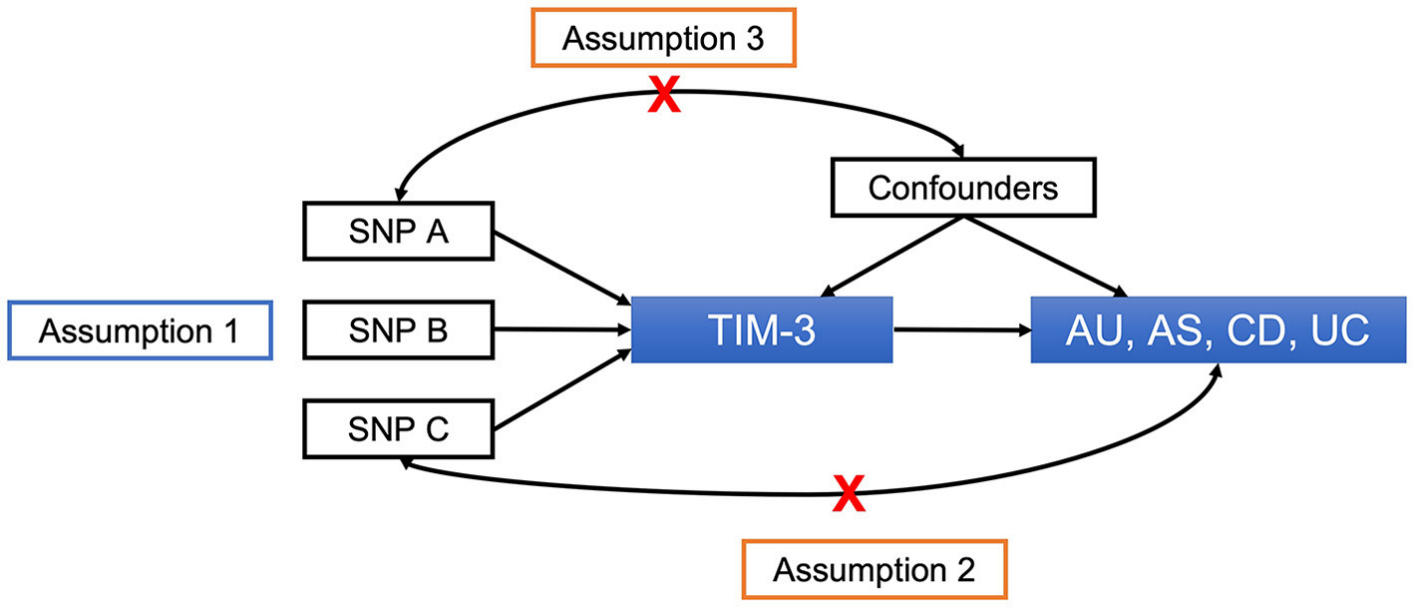
Principles of MR and assumptions required for unbiased causal effect estimation. SNPs, single-nucleotide polymorphisms; *TIM-3*, T-cell immunoglobulin and mucin domain 3; AU, anterior uveitis; AS, ankylosing spondylitis; CD, Crohn's disease; UC, ulcerative colitis.

We employed a two-sample MR design to assess the causal relationships between *TIM-3*, AU, and associated ADs. To obtain a non-biased estimate of the causal effect, three assumptions are required (Figure 1) (24). First, the genetic instrument must be tightly linked to the exposure. Second, there must not be any pathways other than the exposure through which the genetic instrument could influence outcomes. Third, the genetic instrument must not be relevant to confounders of the exposure-outcome relationship.

### Data sources

Summary-level GWAS statistics for the MR analysis were obtained from the MR-Base NHGRI-EBI GWAS catalog (https://gwas.mrcieu.ac.uk/) (25). The *TIM-3* dataset (eqtl-a-ENSG00000135077) was used as the exposure, and it comprised 31,684 participants. To represent a diversity of distinctive AU-associated systemic immune diseases, we selected ankylosing spondylitis (AS), Crohn's disease (CD), and ulcerative colitis (UC) as outcomes: AS GWAS (ukb-a-88) with 968 cases and 336,191 controls, CD GWAS (ukb-a-103) with 1,032 cases and 336,127 controls, and UC GWAS (ukb-b-7584) with 2,439 cases and 460,494 controls. These details are summarized in Table 1.

**Table 1:** Description of GWAS summary statistics of traits.

**Trait**	**GWAS Catalog accession number**	**Sample size**	**Number of SNPs**	**Population**
*TIM-3*	eqtl-a-ENSG00000135077	31,684	19,275	European
Ankylosing spondylitis	ukb-a-88	337,159	10,894,596	European
Crohn's disease	ukb-a-103	337,159	10,894,596	European
Ulcerative colitis	ukb-b-7584	462,933	9,851,867	European

In parallel, AU statistics were obtained from clinical GWAS analysis and used as the other outcome (26); this dataset included 2,752 AS patients with acute AU (cases) and 3,836 AS patients without acute AU (controls), and 7,436,415 single-nucleotide polymorphisms (SNPs) were available.

The participants involved were all of European descent, minimizing the possibility of demographic stratification.

This study used only publicly available data, and the relevant ethical approval can be found in the corresponding studies.

### MR analysis

MR analyses of causal relationships between exposure (*TIM-3*) and outcomes (AU, AS, CD, and UC) were conducted using the TwoSampleMR v0.5.5 package (27). We selected independent genome-wide significant variants as genetic instruments for *TIM-3* by applying the following criteria: (1) The *p*-value on *TIM-3* < 5 × 10^−8^, (2) linkage disequilibrium (LD) *r*^2^ < 0.001, and (3) LD distance > 10,000 kb.

The inverse-variance weighting (IVW) method aims primarily to estimate the role of instrumental variables on an outcome (27, 28), and it can be dependably applied to a single large-scale dataset (29). Consequently, the IVW method was selected as the primary method to estimate the relationships between exposure (*TIM-3*) and outcomes (AU, AS, CD, and UC) in this study.

### Sensitivity analysis

To further probe the stability of our MR analysis results, we conducted an integrated and comprehensive sensitivity analysis using the following methods. Augmented versions of these three methods are available for sensitivity analysis through the TwoSampleMR R package, including MR-Egger regression (30), weighted median method (31), and weighted mode (27), all of which tolerate the existence of horizontal pleiotropy but possess inferior statistical power compared to IVW. Furthermore, we evaluated the robustness of the ascertained associations and the implications of latent horizontal pleiotropy using Egger intercept calculations (32), MR pleiotropy residual sum and outlier (MR-PRESSO) testing (32), leave-one-out analysis (27), and heterogeneity testing. A Bonferroni-corrected *p*-value of 0.0125 (0.05/4) was considered statistically significant.

## Results

**Figure 2:**
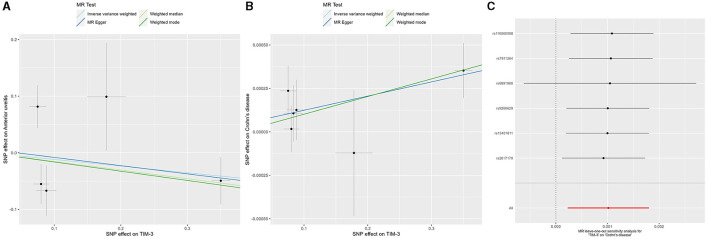
MR analyses of genetic proxy for *TIM-3* levels on risks of anterior uveitis and Crohn's disease. Scatterplot of MR analysis of *TIM-3* and anterior uveitis **(A)** and Crohn's disease **(B)**; the slope of each line represents the causal association for the specified method. **(C)** Leave-one-out analysis for IVW MR of *TIM-3* on Crohn's disease in summary-level analyses. *TIM-3*, T-cell immunoglobulin and mucin domain 3; MR, Mendelian randomization; SNP, single-nucleotide polymorphism; SE, standard error.

The IVW method focuses on exploring the causal relationship between *TIM-3* expression and outcomes. These results showed a protective effect of *TIM-3* for AU although lacking statistical significance (OR = 0.889, 95% CI = 0.631–1.252, *p*-value = 0.5; Table 2). Meanwhile, the MR-Egger, weighted median, and weighted mode methods also suggested that *TIM-3* might play a protective role in AU (OR < 1). Interestingly, *TIM-3* exerted a small facilitative effect on CD (OR = 1.001, 95% CI =1.0002–1.0018, *p*-value = 0.011; Table 2), with this result also supported by weighted median analysis. Scatter plots of the effects of *TIM-3* on AU and CD are shown in Figures 2A, B. However, the causal effects of *TIM-3* on AS and UC could not be elucidated (*p*-value > 0.0125). In summary, these results reveal that *TIM-3* plays a role as a risk factor for CD and might act as a protective factor for AU.

**Table 2:** The causal effect of *TIM-3* on outcomes.

**Outcomes**	**SNPs**	**Method**	**OR**	**95% CI**	***P*-value**
Anterior uveitis	5	Inverse variance weighted	0.889	0.631–1.252	0.500
		MR Egger	0.864	0.454–1.644	0.687
		Weighted median	0.858	0.685–1.075	0.183
		Weighted mode	0.850	0.663–1.089	0.268
Ankylosing spondylitis	6	Inverse variance weighted	1.001	0.9991–1.0026	0.357
		MR Egger	1.000	0.9969–1.0026	0.856
		Weighted median	1.000	0.9997–1.0013	0.237
		Weighted mode	1.000	0.9996–1.0012	0.345
Crohn's disease	6	Inverse variance weighted	1.001	1.0002–1.0018	**0.011**
		MR Egger	1.001	0.9995–1.0021	0.280
		Weighted median	1.001	1.0002–1.0019	0.021
		Weighted mode	1.001	1.0002–1.0019	0.069
Ulcerative colitis	5	Inverse variance weighted	1.000	0.999–1.0009	0.945
		MR Egger	1.001	0.9993–1.0022	0.406
		Weighted median	1.000	0.9992–1.0011	0.753
		Weighted mode	1.000	0.9993–1.0012	0.636

OR, odds ratio; 95% CI, 95% confidence interval.

Significant associations (*P*-value < 0.0125) are bolded.

The robustness of the relationship between *TIM-3* and CD was assessed via comprehensive sensitivity analysis. In the Egger intercept calculation test, the Egger intercept closely approached a value of zero, and the *p*-value was >0.0125 (intercept = 8.24^−5^, *p*-value = 0.798), suggesting the absence of directional horizontal pleiotropy effects. MR-PRESSO testing revealed a lack of horizontal pleiotropic outliers influencing the results (*p*-value of Global Test = 0.151). Additionally, the relationship was unaffected by outliers, as determined by leave-one-out analysis (Figure 2C). The absence of significant horizontal pleiotropy and heterogeneities was further supported by heterogeneity testing (*p*-value of the IVW method: 0.055; *p*-value of MR-Egger method: 0.086). Overall, the results of our sensitivity analysis provide further evidence to support the contribution of *TIM-3* to CD risk.

Furthermore, MR analyses between *TIM-3* and five additional immunologic diseases (systemic lupus erythematosus, psoriasis, rheumatoid arthritis, multiple sclerosis, and juvenile idiopathic arthritis) do not support a causal association of *TIM-3* with the risk of these immune diseases (Supplementary Tables S1, S2).

## Discussion

In this study, we employed a two-sample MR approach to examine the potential causal relationship of *TIM-3* with AU, as well as with three commonly associated systemic immune diseases: AS, CD, and UC. Our data show that *TIM-3* may have a protective effect in AU (OR = 0.889, *P*-value = 0.5 for the IVW method) but could also be a risk factor in CD (OR = 1.001, *P*-value = 0.011 for the IVW method). These intriguing results prompt new questions about the function of TIM-3 in various autoimmune diseases.

Previous research has shown that TIM-3 expression in classical monocytes is significantly reduced in patients with active uveitis, with this reduction persisting after interferon-α2a treatment (33). Our prior study indicates not only that TIM-3 expression is increased during the early stages of ovalbumin-induced ACAID in mice (14) but also that the TIM-3/Galectin (Gal)-9 pathway plays a critical role in maintaining the immune-privileged status of corneal allografts (34). ACAID is considered to be a representative phenomenon linked to the immune privilege (IP) of the eye. Damage to ocular IP is thought to play a significant role in the pathogenesis of uveitis. Different anatomical types of uveitis exhibit varying levels of IP. Inflammation affecting the uvea tract (iritis, cyclitis, and choroiditis) is considered to have a “low” level of IP, while inflammation of the retina (retinitis or retinal vasculitis) demonstrates a “high” level of IP due to the presence of blood-retinal barriers (BRB) (35). In mice, the normal iris-ciliary body only weakly expresses TIM-3 mRNA (34). Based on these findings, we hypothesized that high levels of TIM-3 expression might protect patients from AU attacks by enhancing local IP. Despite the lack of statistical significance for the protective effect of *TIM-3* on AU, all four MR methods employed in our study generated consistent ORs (<1, Table 2; Figure 2A). Future research, including animal experiments and clinical results, will help refine these conjectures further.

Traditionally, pro-inflammatory Th1/Th17 immune responses are hallmark features of uveitis (36), while a Th2 response characterized by high levels of IL-4, IL-5, and IL-13 is associated with UC (12). Recent studies have suggested that enhanced Th17 cell function is correlated with disease activity in UC (19). Blocking or dysregulating TIM-3 has been shown to exacerbate imbalanced Th cell activity, both enhancing Th1/Th17 function and impairing Treg function (7). TIM-3 and TIGIT have been shown to be enhanced on Notch/STAT-3-co-stimulated CD4^+^ T cells (37). Notch/STAT-3-driven Blimp-1/c-Maf axis is a common anti-inflammatory pathway in human CD4+ T cells, which was defective in Crohn's disease patients. Co-inhibitory receptors, such as LAG-3, CD49b, PD-1, TIM-3, and TIGIT, were found co-expressed by IL-10-producing Tr1 cells, which correlate with their suppressive capacity (38). These results suggest that TIM-3 may play an important role in the inflammatory pathogenesis of IBD.

An interesting finding has emerged regarding the differential action of TIM-3 in two types of IBD. Our present study suggests that *TIM-3* is a risk factor for CD but has no effect on UC (OR = 1.000, *P*-value = 0.945 for the IVW method). Previous studies have demonstrated that mRNA levels of TIM-3 are decreased in Th cells isolated from both PBMCs (naïve or stimulated) and the intestinal mucosa of patients with CD, but these effects are not observed in ulcerative colitis patients (18, 19). Furthermore, a significant difference in TIM-3 expression levels is evident after infliximab therapy (18). Recently, Sun et al. (39) found that two *TIM-3* variants (rs1036199 and rs10515746) are significantly associated with an increased risk of penetrating diseases in patients with CD. Another *in vitro* study indicated that monocytes from active UC patients express TIM-3 at relatively low levels, which decrease further following stimulation with autologous fecal bacteria (40). Gal-9 significantly suppresses the expression of TNF-α and IL-6 in monocytes, and this suppression is directly correlated with TIM-3 expression levels (40). However, there are not enough studies available to fully explain the probable different roles of TIM-3 among different autoimmune diseases. Further studies are warranted to investigate the mechanisms underlying these associations.

Our study has some limitations. First, all analyses were conducted on participants of European ancestry, so our results may not necessarily be generalizable to people of other racial or ethnic backgrounds. Second, the magnitude of the effect in this study is relatively small. Although we demonstrated that an inherited *TIM-3* level is associated with an increased risk of CD, the exact mechanism remains unclear. Third, for the AU analyses, we selected AS patients with acute AU as cases and AS patients without AU as controls. This may imply the presence of selection bias that could have affected our results.

In conclusion, our MR study suggests that genetic predisposition to higher *TIM-3* expression levels increases the risk of CD, while lower *TIM-3* levels might have a protective effect against AU. Our results provide new insights regarding the roles of TIM-3 in systemic autoimmune diseases and highlight critical directions for future research.

## Data availability statement

The datasets presented in this study can be found in online repositories. The names of the repository/repositories and accession number(s) can be found below: https://gwas.mrcieu.ac.uk, eqtl-a-ENSG00000135077; https://gwas.mrcieu.ac.uk, ukb-a-88; https://gwas.mrcieu.ac.uk, ukb-a-103; https://gwas.mrcieu.ac.uk, ukb-b-7584.

## Ethics statement

Ethical review and approval was not required for the study on human participants in accordance with the local legislation and institutional requirements. Written informed consent for participation was not required for this study in accordance with the national legislation and the institutional requirements. Written informed consent was not obtained from the individual(s) for the publication of any potentially identifiable images or data included in this article.

## Author contributions

YQW, MLG, FFL, and DL contributed to the study design. DL and RCZ contributed to data collection and analysis. DL, RCZ, and CT wrote the manuscript. All authors contributed to the article and approved the submitted version.
