# Supplementary material for: Association of TIM-3 with anterior uveitis and associated systemic immune diseases: a Mendelian randomization analysis
Source: Front Med (Lausanne). 2023 Jun 15;10:1183326. doi: 10.3389/fmed.2023.1183326 (PMC10313383; doi:10.3389/fmed.2023.1183326)
Supplement: Supplementary file 1 [file Table_1.DOCX]

Table S1. Description of GWAS summary statistics of five additional traits

| **Trait** | **GWAS Catalog**  **accession number** | **Sample size** | **Number of SNPs** | **Population** |
| --- | --- | --- | --- | --- |
| **Systemic lupus erythematosus** | ebi-a-GCST003156 | 14,267 | 7,071,163 | European |
| **Psoriasis** | ukb-b-10537 | 462,933 | 9,851,867 | European |
| **Rheumatoid arthritis** | ukb-b-11874 | 463,010 | 9,851,867 | European |
| **Multiple sclerosis** | ukb-b-17670 | 462,933 | 9,851,867 | European |
| **Juvenile idiopathic arthritis** | ebi-a-GCST005528 | 15,872 | 103,767 | European |

Table S2. Causal effect of TIM-3 on five additional outcomes.

| **Outcomes** | **SNPs** | | **Method** | **OR** | **95% CI** | ***P*-value** |
| --- | --- | --- | --- | --- | --- | --- |
| **Systemic lupus erythematosus** | 6 | Inverse variance weighted | | 1.060 | 0.8574-1.3115 | 0.589 |
|  |  | MR Egger | | 0.997 | 0.6798-1.4625 | 0.989 |
|  |  | Weighted median | | 1.039 | 0.9039-1.1941 | 0.591 |
|  |  | Weighted mode | | 1.028 | 0.8896-1.1875 | 0.725 |
| **Psoriasis** | 5 | Inverse variance weighted | | 0.999 | 0.9916-1.0070 | 0.861 |
|  |  | MR Egger | | 1.000 | 0.9863-1.0146 | 0.962 |
|  |  | Weighted median | | 0.999 | 0.9974-1.0001 | 0.075 |
|  |  | Weighted mode | | 0.999 | 0.9979-1.0010 | 0.547 |
| **Rheumatoid arthritis** | 3 | Inverse variance weighted | | 0.999 | 0.9986-1.0001 | 0.106 |
|  |  | MR Egger | | 1.000 | 0.9985-1.0009 | 0.695 |
|  |  | Weighted median | | 0.999 | 0.9987-1.0002 | 0.127 |
|  |  | Weighted mode | | 0.999 | 0.9987-1.0002 | 0.307 |
| **Multiple sclerosis** | 4 | Inverse variance weighted | | 1.001 | 0.9978-1.0044 | 0.512 |
|  |  | MR Egger | | 0.999 | 0.9937-1.0050 | 0.837 |
|  |  | Weighted median | | 1.001 | 0.9998-1.0014 | 0.173 |
|  |  | Weighted mode | | 1.000 | 0.9996-1.0013 | 0.386 |
| **Juvenile idiopathic arthritis** | 2 | Inverse variance weighted | | 0.243 | 0.0400-1.4813 | 0.125 |
